# Coloration on Bluish Alginate Films with Amorphous Heterogeneity Thereof

**DOI:** 10.3390/polym15173627

**Published:** 2023-09-01

**Authors:** Soo-Yeon Yang, Dong-Soo Kang, Chang-Yull Lee

**Affiliations:** 1 Institute of Aerospace System, Inha University, Incheon 21999, Republic of Korea; 2 Department of Materials Science and Engineering, Inha University, Incheon 22212, Republic of Korea; dskang1266@inha.ac.kr; 3 Department of Aerospace Engineering, Inha University, Incheon 21999, Republic of Korea

**Keywords:** alginate, iridescence, structural coloration, micro-spacing, self-assembly, hydrogel

## Abstract

Using sodium alginate (Alg) aqueous solution containing indigo carmine (IdC) at various concentrations we characterized the rippled surface pattern with micro-spacing on a flexible film as intriguing bluish Alg–IdC iridescence. The characterization was performed using Fourier-transform infrared spectroscopy, ultraviolet–visible spectroscopy, field emission scanning electron microscopy, atomic force microscopy, electron microscopy, differential scanning calorimetry, thermogravimetric analysis, X-ray diffraction analysis, and photoluminescence detection. The edge pattern on the film had a maximum depth of 825 nm, a peak-to-peak distance of 63.0 nm, and an average distance of 2.34 nm. The center of the pattern had a maximum depth of 343 nm and a peak-to-peak distance of 162 nm. The pattern spacing rippled irregularly, widening toward the center and narrowing toward the edges. The rippled nano-patterned areas effectively generated iridescence. The ultraviolet absorption spectra of the mixture in the 270 and 615 nm ranges were the same for both the iridescent and non-iridescent film surfaces. By adding Ag^+^ ions to Alg–IdC, self-assembled microspheres were formed, and conductivity was improved. Cross-linked bluish materials were immediately formed by the addition of Ca^2+^ ions, and the film was prepared by controlling their concentration. This flexible film can be used in applications such as eco-friendly camouflage, anti-counterfeiting, QR code materials for imaging/sensing, and smart hybrid displays.

## 1. Introduction

Visual perception in humans involves the interpretation of light signals in the brain. When light is reflected, diffracted, scattered, or refracted from the surface of an object, it stimulates the photoreceptors of the visual tissue and is transmitted to brain tissue via visual nerves. The perception process from widespread structural coloration results from the surface array of an object. In general, the process of color perception in nature involves the perception of light reflected from various surfaces of color sources. Furthermore, light striking the micro- or nano-surface of an object spatially with a nano-lattice structure is diffusely reflected based on its arrangement characteristics in nature. Thus, the structural colors show that the light is mixed with the secondary colors in visible light of the light source based on the direction of the light, and finally, the light appears as white light on a nano-surface at viewable angles. Some species use body color for information exchange and protection by cleverly combining dye composition, arrangement, and background to achieve color changes depending on conditions.

Following the introduction of variable structural colors to natural organisms, many researchers have focused on artificial variable coloration materials, including biologically inspired designs, biomimetic materials, and techniques to produce variable coloration under natural light. Biomimetic coloration, which can be reversibly changed, is receiving increasing attention for application in display materials. Elaborate nanostructures inspired from natural organisms are of significant interest to scientists because these can be implemented close to natural colors without a large power source.

The applications of bioinspired photonic components, natural polymers, hybrid hydrogel films, core–shell particles, anisotropic Janus particles, etc., have been reported using various methods imitating nature, such as butterflies, beetles, hummingbirds, and chameleons. These structural color materials exhibit properties related to adhesion, tenability, and elasticity of the arrayed media, particularly as the optical wavelength range varies with particle size, composite content, and alignment [1,2,3,4,5,6].

In tropical shade-adapted plants, blue reflection from a multilayer structure enhances photosynthetic light capture while minimizing photodamage. Blue leaf iridescence at wavelengths of 400–485 nm protects tissues against the inhibition of photosynthesis from excessive irradiance and photodamage by reducing light absorption [7]. With advances in science and technology, optical signal transmission and display development have begun to achieve the same effect as that of the natural light in various areas at low cost and with high efficiency. Furthermore, attempts have been made to detect the surface color of soft objects, such as feathers of a bird or scales of an aquatic organism, whose colors vary based on the condition of natural light. The epidermal layers maintain the micro-regularity of striations around us, such as the natural microstructure of Tulipa purple crystal petals, Favonius taxila, and magpie plumage. Fernandes et al. [8] reported that the mechanical and optical properties of thin cellulosic films could be tuned via nanorods. The low-cost cellulosic material appears to be a biomimetic structure similar to the type of grating observed in tulip petals. Iridescence and coloration are produced by coherent light scattering during film interference. Diffraction gratings consisting of a series of aligned and precisely spaced running reflective surfaces can produce iridescence.

Interestingly, Wu et al. [9] showed that a monolayer of microspheres on the adhesive side of a transparent tape and an interferometric structure on the surface of polystyrene microspheres could lead to structural color-induced non-iridescence/iridescence under coaxial/non-coaxial illumination and viewing conditions. Deng et al. [10] introduced programming information using the phase-change materials based on their thermal and optical properties without the need for any sophisticated equipment.

In addition to research on organic materials, Wu and Hong reported dopamine–melanin nano-film reflectors fabricated on a substrate by mimicking the bilayer structure of the keratin layer and melanin granules, such as that in bird plumage. The biomimetic coloration depended on the granule size and film thickness of 50–200 nm obtained by controlling the concentration of the dopamine solution and reaction time [11]. As a part of cutting-edge research, Miyamoto reported that a structural color gel of perovskite nanosheets exhibited a reversible mechanochromic fast response with high sensitivity [12].

Intriguingly, the ellipsoidal or mixed core–shell melanin particles have been studied for structural color applications. As the aspect ratio of the elliptical particles increased, the reflection wavelength of the assembled sample blue-shifted depending on the minor axis of the particle [13,14].

Recently, Wang et al. [15] fabricated two-dimensional self-assembled colloidal crystals based on polystyrene with nano- to micro-sizes spanning two orders of magnitude on challenging substrates, including hydrophobic, rough, curved, and structured microchannels. The capillary transfer method has high versatility, quality, and simplicity.

Inspired by spatiotemporal icing technology, Miao et al. [16] introduced freeze-photopolymerization to construct inverse structural color hydrogels with micro/nano-multiscale structures. These materials exhibit designable morphological and heterogeneous structural color modulation. Furthermore, because of the squeezing effect of the growing ice crystals, the spacing between the surface-charged nanoparticles decreases, resulting in a blue shift.

In another study, Zhou et al. [17] demonstrated the structural coloration of self-organized wrinkled thin films via spatially arbitrary preparation and reversible regulation of ultraviolet to near-infrared irradiation using light-sensitive polymers. 

The present study focused on expressing iridescent structural coloration to realize natural biomimetic light using eco-friendly materials. Following the demonstration of tunable structural coloration in organisms, several studies have focused on artificial tunable coloration with photonic materials, including biologically inspired designs, biomimetic materials, and techniques to produce materials with effects close to those of natural coloration.

We developed biologically inspired materials for structural coloration. Notably, these materials are scientifically innocuous ingredients. Sodium alginate (NaC_6_H_9_O_7_; Alg) is a natural hydrophilic linear polysaccharide used in eco-friendly applications owing to its biocompatible and biodegradable amphiphilic characteristics. The ionic and hydrophilic components of sodium alginate increase its solubility in water [18,19]. Alginate cross-linking occurs in the presence of divalent ions in solution because of their specific structures. In particular, the anionic moieties readily bind with most divalent cations and various composites and have gel-oriented properties upon assembly. Therefore, it can be used as a porous scaffold with cell permeability by absorbing water and cross-linking it to become a component of functional tissues [20,21,22].

Alg isolated from sea wood has been widely used as a biomaterial in tissue engineering because of its easy ionic gelation, biocompatibility, and tunable rheological and physical properties. The gelation response and tunable viscosity of alginates contribute to their promising printability, particularly for extrusion bioprinting. Alg has been used as a primary bio-ink material for bioprinting myoblast-laden structures [23].

In addition, natural amphiphilic hydrogels such as polymers, biomaterials utilizing melanin, proteins, polysaccharides, porphyrins, and collagens are stimulating a novel idea in bioelectronics, ionics, displays, and devices development [24,25].

A study by Holtz and Asher provided inspiration for chemical sensing materials that diffract at visible wavelengths by adjusting a crystalline colloidal array of polymer spheres 100 nm in diameter within hydrogels and binding a molecular recognition group with crown ethers for metal ions to produce specific colors [26]. Polymer-assisted photochemical silver-deposition-based color printing is effective on a wide range of substrates and offers ideas for a variety of future applications, from security labeling to color displays [27]. The studies on mechanochromic elastomer are still in progress [28].

In this study, iridescent colors were implemented by introducing a ripple film surface that can prevent internal damage even with strong ultraviolet rays. Low concentrations of naturally occurring indigo carmine (IdC) were added to 5 wt% and 10 wt% Alg aqueous solutions to limit the range of coloration, as in shade plants. By adding Ag^+^ and Ca^2+^ ions to Alg–IdC, self-assembled microspheres were formed, the color was tuned, and the conductivity was improved. It is a simple method and material that can be used to achieve the properties of electronic inks as reported and reviewed by Han and Dong et al. [29] and Eshkalak et al. [30], respectively.

As for protection against excessive irradiance and photodamage in plants, our bluish iridescent materials were characterized by a simple method on a substrate with the rip-pled microsurfaces of eco-friendly mixtures of varying concentrations. The spacing pattern widened toward the center and narrowed toward the edges. The rippled micro-patterned areas effectively generated iridescence. These features of structurally colored materials indicate their potential value for mimicking structurally colored organisms in fields such as light regulation, temporal camouflage, and biomimetic technology. Furthermore, we believe that tunable and high-potential materials will be useful for secure communication, information storage, color-coded signals, bioprinting for optics, smart displays, imaging/sensing materials, smart hybrid hydrogel coatings, tissue engineering, construction of intelligent sensors, anti-counterfeiting devices, biomedical analysis, optical devices, information safety, and bio-electrolyte indicators.

## 2. Materials and Methods

### 2.1. Materials and Instrumentations

All chemical reagents used in experiments were of analytical grade and were used as received, without further purification. The Alg (CAS No. 9005-38-3), IdC (CAS No. 860-22-0), silver nitrate (AgNO_3;_ CAS No. 7761-88-8), and calcium chloride (CaCl_2;_ CAS No. 10043-52-4) were purchased from Sigma-Aldrich Co. Ethyl alcohol (EtOH; CAS No. 64-17-5) and distilled water (DW; CAS No. 7732-18-5) were purchased from Thermo Fisher Scientific.

X-ray diffraction (XRD) patterns were recorded by a Philips-X’Pert Pro Diffractometer (Philips, Amsterdam, The Netherlands), X-ray diffractometer using Ni-filtered Cu Ka radiation. Attenuated total reflection and Fourier-transform infrared (FT-IR) spectra were recorded on Bruker VERTEX 80V (Bruker, Hamburg, Germany) or Shimadzu IRAffinity-1S (Shimadzu, Tokyo, Japan). Electronic spectra of the samples were recorded using a Perkin-Elmer LS-55 luminescence spectrometer (PerkinElmer, Waltham, MA, USA). Field emission scanning electron microscopy (FE-SEM) images were obtained using the LEO-1455VP instrument equipped with an energy dispersive X-ray spectroscope. The electronic spectra of the complexes were recorded on a Thermo Scientific^TM^ UV–Vis scanning spectrometer (Model Evolution 220, Carlsbad, CA, USA) and a microscopic images smart high-resolution stereo microscope (Smartzoom 5, Hamburg, Germany). Differential scanning calorimetry (DSC) was performed using a NETZSCH DSC 200F3 instrument (NETZSCH Premier Technologies, Tokyo, Japan). The main sensor used was a Ceramic-FRS5. Samples were heated at a scanning rate of 10 K/min at temperatures ranging from 20 to 350 °C. Aluminum pans and lids were used as references for all samples, and the analyses were performed under a nitrogen flow of 50 mL/min. Subsequently, energy calibration was performed. The melting point was measured as the peak temperature (tm). Thermogravimetric analysis (TGA) was performed using a TG 209 F3/ NETZSCH (Japan). Atomic force microscope (AFM) images were measured with a Bruker Nanoscope Multimode IVa (Bruker, Hamburg, Germany) with a vertical resolution of 0.1 Å, a lateral resolution of 1 Å, and a scan size (Lateral X, Y Range) of 125 µm × 125 µm × ±5.0 µm. The conductivity of samples was tested using a DAQ6510 DATA Acquisition/multimeter system from Keithley offering measurement ranges 10 pA to 10 A and 1 mΩ to 100 MΩ.

### 2.2. Preparation of Bluish Materials and Ion Complexations

**Figure 1:**
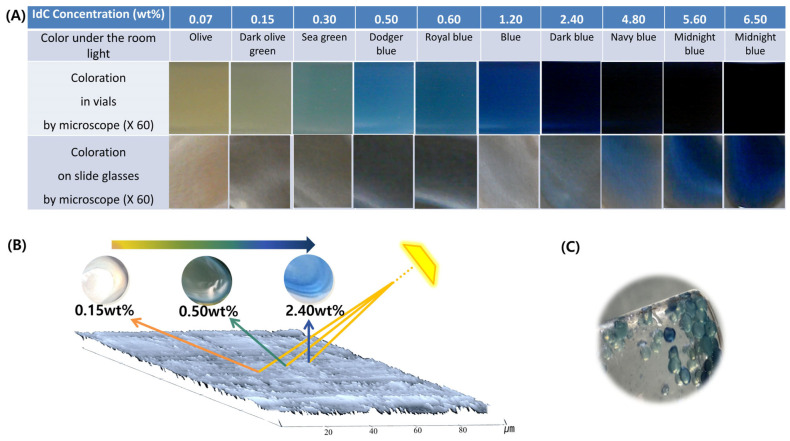
(**A**) Coloration of 5 wt% Alg aqueous solution with saturated IdC (aq.) based on corresponding ratio under the room light. (**B**) Iridescence revealed by concentration gradient Alg–IdC under sunlight. (**C**) Structural coloration of calcium alginate salt containing 2.4 wt% saturated IdC.

First, 1.0 g and 0.5 g Alg were sufficiently dissolved in DW to adjust the total weight to 10 g over 24 h. Next, 0.11 g IdC was dissolved in 10 g DW to saturate the solution. Then, total weight of Alg–IdC mixture was fixed to 5 g by adding 0.12 g (a drop) of saturated IdC (aq.) to 4.88 g viscous 5 wt% Alg aqueous solution to achieve a IdC concentration of 2.4 wt% in the Alg–IdC mixtures. The following various concentrations (Figure 1A) were adjusted via changing the ratio of IdC and Alg solution usage. The pre-mixtures were then vortexed and stabilized for more than 12 h at ambient temperature. Next, 0.01 N AgNO_3_ (aq.) solution and saturated IdC (aq.) solution were mixed for 2 h. The IdC (aq.) solution mixed with 0.01 N silver ions (aq.) or 0.01 N calcium ion (aq.) was slowly added to the 5 wt% Alg aqueous solution, followed by stirring the solution for 2 h at ambient temperature to attain ion complexation.

### 2.3. Processing of Iridescent Films

Mixtures with specific concentrations were prepared as previously described. The 5 wt% Alg–IdC mixture, 0.5 g, was transferred onto a 2 cm × 2 cm quartz substrate. Subsequently, the film was moderately dried on a hotplate at 40 °C for 2 h to remove water and volatile residues from the surface.

## 3. Results and Discussion

### 3.1. Coloration by the Ratio of Alg–IdC Aqueous Solution

**Figure 2:**
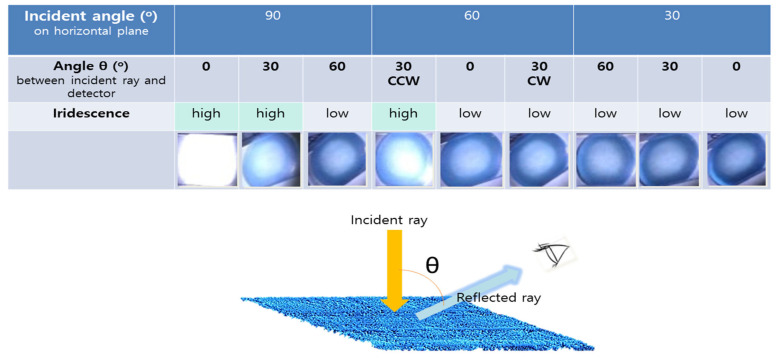
Iridescence on Alg–IdC films with rippled surfaces based on the incident ray.

The hue of Alg–IdC was changed to various concentrations by changing the IdC to 5 wt% Alg (aq.), as shown in Figure 1. The hue, owing to the gradient in the Alg and IdC concentration ratio, changed from olive green, sea green, dodger blue, or navy blue to midnight blue. The rippled surface of the glass slide appeared to be iridescent under sunlight. The range of concentration to coloration was 0.1–4.8 wt% saturated IdC (aq.) to 5 wt% Alg (aq.). Figure 2 shows the angular iridescence of a dry film fabricated with 1.00 ± 0.01 g Alg–IdC, ~2.5 cm in diameter, and ~70± 5 µm in thicknesses on glass slides or quartz (Figure 1).

### 3.2. Iridescence on Alg–IdC Film Based on Tilt Angle

As shown in Figure 2, iridescence differences were observed in the rippled Alg–IdC film based on the incident ray. When the ray was incident at 90° to the film, luminance was high with iridescence. Iridescence persisted at θ = 30° between the incident ray and detector; however, a larger θ resulted from the attenuation of iridescence. When the ray was incident at 60° to the film, iridescence with the high luminance appeared at θ = 30° counterclockwise. In other directions, the luminance decreased, as did iridescence. When the ray was incident at 30° to the film, the film reflection color was considered its own color and was faintly iridescent. Thus, the effect of iridescence was high within an angle of 30° between the incident ray and detector.

We intended to detect iridescent changes in the angle-dependent reflection spectrum owing to the viewing conditions between the incident light and detector. Intriguingly, according to a study by Fan et al. [4], a red shift in the visible region is observed under non-coaxial illumination when various incidence angles are increased. Although slightly different from our objective, this finding was supported by the conclusion that the reflected ray on the rippled film had a boundary region where the iridescent luminance was able to increase (see Supplementary Videos S1 and S2 on iridescence under light).

### 3.3. Characterization of Alg–IdC Films

**Figure 3:**
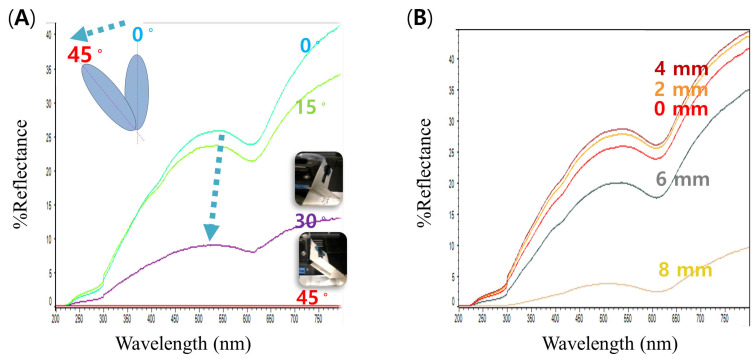
UV–vis spectra of Alg–IdC. (**A**) UV–vis reflectance at 5 wt% Alg–IdC. The wavelength shifted owing to the tilt angle of plate. (**B**) UV–vis reflectance spectra as a function of distance from the center.

The light-responsive behavior of the surface of Alg–IdC films prepared from solutions was investigated using the UV–vis absorption spectroscopy (see Figure 3). The reflectance of film surface tended to decrease as the tilt angle increased. When the plate was vertical, the absorption wavelength was 545.5 nm, and it dispersed, decreasing to 531 nm at a tilt angle of 30°. This result shows that the absorption wavelength tends to slightly blue-shift as the Alg–IdC plate is tilted (see Figure 3A). Considering the change in reflectance for each radial distance from the center of the film, the reflectance increased up to 4 mm from the center and decreased beyond 4 mm. This is considered to be a variation in the thickness of the film, increasing up to 4 mm from the center of the rippled film and thinning outward (Figure 3B).

**Figure 4:**
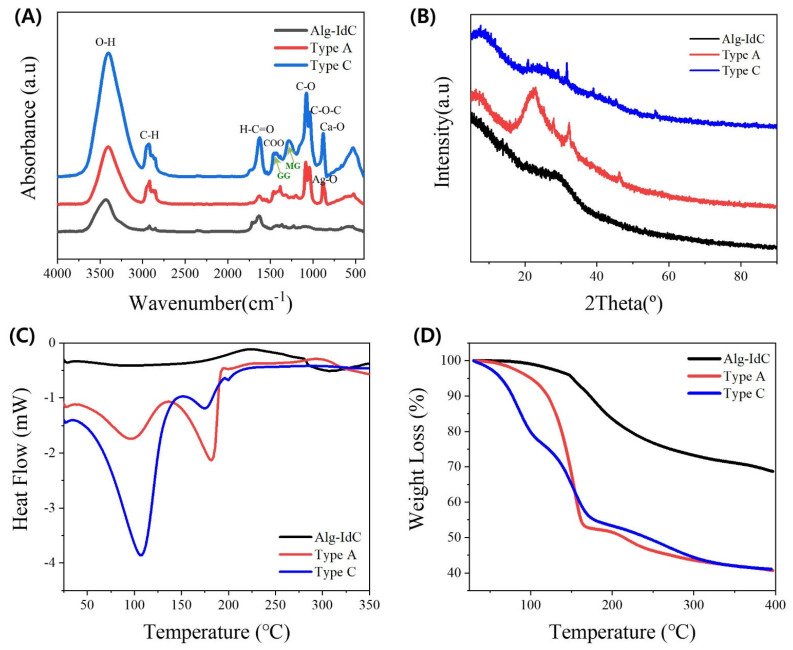
Characterization of Alg–IdC, Type A (Alg–IdC containing Ag^+^), and Type C (Alg–IdC containing Ca^2+^): (**A**) FT-IR spectra of 5 wt% Alg–IdC (black line), Type A (red line), and Type C (blue line); (**B**) XRDs of Alg–IdC, Type A, and Type C; (**C**) DSCs of Alg–IdC, Alg–Ag, Type A, and Type C; and (**D**) TGA of Alg–IdC, Type A, and Type C.

**Scheme 1:**
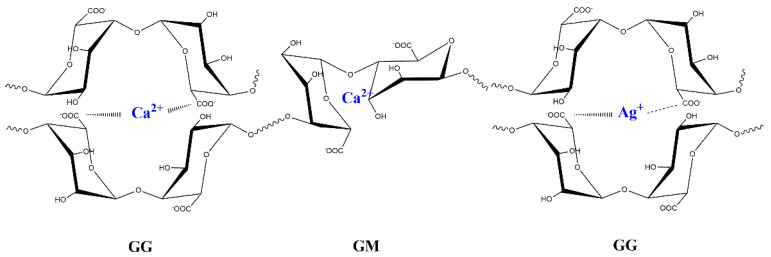
Possible interaction of Alg with Ca^2+^ or Ag^+^ ions assembled inside the hydrogel.

As depicted in Figure 4A, the FT-IR spectra of the 5 wt% Alg–IdC film exhibited a stretching vibration peak at 3409 cm^−1^ for its hydrogen-bonded OH group, the peak at 2930 cm^−1^ was correlated with the stretching vibrations of aliphatic –CH, and the peak at 1350 cm^−1^ corresponded to the alkyl –CH bending vibration. This was because the IdC content was negligible for Alg, with an Alg:IdC ratio of 1:0.00576 in 5 wt% Alg solution. Asymmetric and symmetric stretching of the H-C=O group of Alg was observed at 1642 and 1401 cm^−1^, respectively. In addition, Alg showed characteristic peaks at 1080 and 820 cm^−1^ corresponding to the C–O stretching vibration and Na–O bond vibration, respectively, indicating that Alg comprised many networks. For the FT-IR curve of Type C, the stretching vibration of OH was observed at 3402.3 cm^−1^, aliphatic –CH at 2920 cm^−1^, and the alkyl –CH bending vibration at 1384.8 cm^−1^. The cross-linking process with Ca^2+^ resulted in an apparent shift in the –COO^-^ asymmetric stretching vibration from 1642 to 1627.9 cm^−1^ and in the C-O from 1080 to 1085.9 cm^−1^. A synchronous –COO^-^ symmetric stretching vibration from 1401 to 1420 cm^−1^ clearly shows the formation of a Type C compound by ionic bonding between the carboxyl groups of Alg and Ca^2+^ at 881.4 cm^−1^. The FT-IR spectra of the Type A compound showed that no new bonds were formed when compared with Type C. Although the Type C showed both GG (G is an abbreviation for the guluronate form of alginate, and GG means the interaction between guluronates) at 1463.9 cm^−1^ and MG (M indicates the mannuronate form of alginate) blocks of –COO^-^ broadly at 1249.8 cm^−1^, Type A showed only GG blocks at 1464 cm^−1^. The C-O-C stretching was observed at 1043.4 cm^−1^ in Type C and at 1039.6 cm^−1^ in Type A. Similar to Ca^2+^, the interaction of the Ag^+^ ion with –COO^-^ was also verified at 881 cm^−1^ [31,32,33,34,35]. The FT-IR results helped to elucidate the chemical structure of complexes formed by cross-linking or coordinating between natural polymers and metal ions inside the hydrogel owing to the interaction of metal ions such as Ca^2+^ or Ag^+^ ions, as shown in Scheme 1 [36,37,38,39].

As shown in Figure 4B, the XRD results of Alg–IdC show an amorphous pattern because it is a mixture of both organic Alg and IdC. Type C cross-linked by activated Ca^2+^ exhibited a similar amorphous pattern, with 2θ detected at 9.3, 20.86, and 31.66 and their corresponding d-spacings being 9.55, 4.25, and 2.82 Å. Type A had a crystal-like pattern with 2θ of 21.6, 23.1, 27.5, 32.32, 33.1, and 46.18 with d-spacings of 4.11, 3.84, 3.23, 2.77, 2.70, and 1.96 Å, respectively. As shown in the 2θ of 32.32 and 46.18, the patterns were partially similar to the face-centered cubic (fcc) Ag nanoparticle colloid structure on the SiO_2_ substrate. Owing to the anionic-rich alginate environment, Ag^+^ ions were considered as the reductive modes inside Type A complexes [34].

Figure 4B shows the XRD pattern for Type A obtained for Alg–IdC containing the silver nanoparticles. Several Bragg reflections were verified, which could be indexed based on the fcc structure of the silver nanocrystals. The XRD pattern suggested that Type A contained the nanoparticles of Ag^+^ ions, showing a crystal-like pattern.

As is evident from Figure 4C and Table 1, the analysis of the DSC results indicated the following. The transition of Alg was initiated at 93.7 °C, induced by thermal self-assembly through chain interactions. The glass transition with endothermic enthalpy of ΔH = 90.82 J/g occurred at *Tg* of 120.9 °C. The Tc of Alg was 161.1 °C, and the phase transition completed at 149.9 °C with exothermic enthalpy of ΔH = −81.8 J/g. The transition of the Alg–IdC film started at 98 °C and was followed by a glass transition at *Tg* of 121.5 °C with endothermic enthalpy of ΔH = 71.71 J/g. The crystallization temperature was Tc 163.9 °C, and the phase transition was completed at 150.8 °C, resulting in exothermic enthalpy of ΔH = −79.14 J/g. The thermal characteristics of Alg–IdC and Alg showed similar behaviors owing to the absolute amount of Alg, as is evident from the data in Table 1. However, for Type A and Type C, the exothermic characteristics appeared in two stages. In terms of chemical structure, the cross-linking by metal ions was attributed to rapid heat transfer in the first step. The decomposition of the Alg main chain was considered to be confirmed in the second step at 175 °C or more. The calcium ions arranged the structures more favorably for heat transfer than the array of silver ions. Therefore, the calorific value was relatively large in the first step.

In the TGA shown in Figure 4D, both Alg and Alg–IdC lean gradually toward thermal degradation after 161 °C. Regardless of the IdC content in the mixture, the thermal results were similar to those of the thermal properties of Alg, which was the main component.

In the graph results, there was no difference in thermal analysis data between Alg and Alg–IdC, so only one of the two was marked in the graph trend.

**Figure 5:**
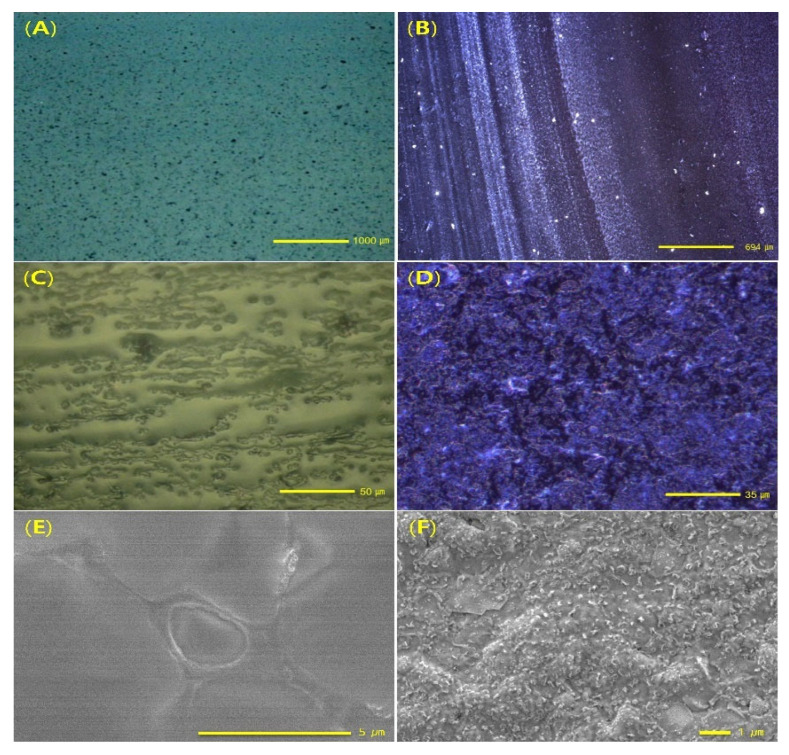
Images of Alg–IdC film. (**A**) Magnification of microscopic image ×100 and (**B**) image (×101, 3526 × 2644 μm^2^) of dense micro-spaced lines obtained by Smartzoom 5 with exposure time of 38.38 ms and resolution of 0.1112 μm/pixel. (**C**) Microscopic, corrugated images at magnification of ×1200. The blue-rippled surface of the sample film is shown as greenish in (**A**) and (**C**) owing to the light source of the microscope. (**D**) Magnified corrugated image (×2000, 178 × 133 μm^2^) shown under a microscope (**B**). SEM images show the film surfaces (the type of grating ×10,000) of Alg–IdC corresponding to (**E**) 0.24 wt% and (**F**) 5.6 wt% IdC ratios, respectively.

Microscopic and SEM images for various concentrations of the rippled Alg–IdC surfaces are shown in Figure 5. Regarding images of the Alg–IdC film, as shown in Figure 5A, the image is at magnification and Figure 5B, the image with dense micro-spaced lines were verified by microscope SMARTZOOM 5 and revealed the mixture surface with heterogeneity and the rippled pattern. In Figure 5C, the blue-rippled surface of the sample film shows the ripple in detail, and the blue-color appears as greenish in Figure 5A and Figure 5C owing to the light source of the microscope. Figure 5D is also an image of rippled film magnified at ×2000 taken under the microscope in Figure 5B. The SEM images show the film surfaces (the type of grating ×10,000) of Alg–IdC corresponding to 0.24 wt% and 5.6 wt% IdC ratios (Figure 5E and Figure 5F), respectively. This is a list of images of rippled surfaces magnified under a microscope. At low magnification, only scattered components are visible, but at higher magnifications, ripples on the surface are visible.

**Figure 6:**
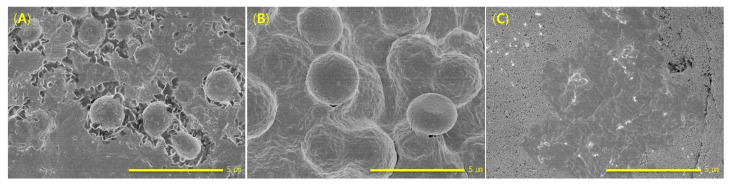
SEM images showing film surfaces (the type of grating ×10,000): (**A**) Type A; (**B**) Type A treated with EtOH, shown as Alg–Ag microsphere-washed IdC; and (**C**) Type C.

SEM analysis was performed to investigate the formation of Type A microspheres from Alg–IdC and Ag^+^ ions by self-assembly. Ag^+^ interacted with Alg chains as seeds for self-assembly, and Alg microspheres containing Ag^+^ interacted with IdC on the self-assembled surface (Figure 6A). Because Alg and IdC are insoluble in EtOH and tend to shrink, when Type A was dropped into H_2_O:EtOH (v/v = 5:1) to induce self-assembly of microspheres, Alg with Ag^+^ formed IdC-washed microspheres (Figure 6B). Therefore, IdC moieties appeared on the Alg surface, and Ag^+^ ions were considered to interact chemically with the carboxylate moieties in the Alg chain to form complexes and self-assemble. Although the conductivity of the rippled film surface was evaluated by increasing the content of Ag^+^ ions in the Alg–IdC film, from the point of view of the Type A film preparation, the optimized 0.01 M concentration of Ag^+^ ions was, approximately, in line with the ionic solubility derived from the solubility product constant of silver sulfate (${Ksp}_{Ag2SO4}=1.2\times 10^{-5}$).

When Ca^2+^ ions were dropped onto Alg–IdC (aq.), Alg was reactive toward Ca^2+^ ions, and Alg was immediately cross-linked to form Type C with a dense structure (Figure 6C). Ca^2+^ is readily used as a cross-linker to form GG or GM of alginates [33]. For Type C, heterogeneous white spots, i.e., insoluble calcium salts from IdC or carboxyl groups, randomly appeared. The dense surface of Type C showed orthogonal-like aggregation during cross-linking. The bond between the Ca^2+^ and Alg structures appeared to be more robust, similar to an ionic complex, than the coordination complexation of Alg with Ag^+^ ions. Therefore, the Type A hydrogel can form microspheres during self-assembly. In tissue engineering, cross-linkable calcium alginate is used to regenerate bones, cartilage, and muscles. As there is a report that cartilage tissue is regenerated with properties similar to those of the actual cartilage, Type C also shows the dense cross-linking of calcium alginate [35,36,37,38,39].

**Figure 7:**
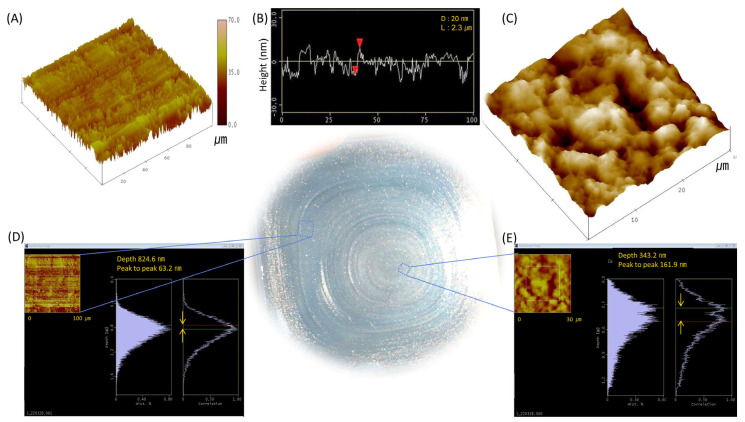
AFM images: (**A**) 3D topographical phase (tapping-mode) image 100 × 100 μm^2^ (x 20 μm/div, z 70 nm/div) of top surface of rippled film prepared from 5 wt% Alg–IdC. (**B**) Section analysis of rippled film surface (x 100 μm, y 70 nm). (**C**) Height tapping-mode image 30 × 30 μm^2^ (x 10 μm/div, z 2 μm/div) of the top surface of film shown in (**A**). (**D**,**E**) Pixel height distribution analyses. AFM tapping-mode image of the top surface of 5 wt% Alg–IdC thin rippled film with a height profile along the line (y 2.0 nm). Scan size in (**D**) is 100 μm and in (**E**) is 30 μm.

To further investigate the pattern structures on the iridescent film, the surface of the Alg–IdC film was investigated using AFM at the micro-and nano-scales. As shown in Figure 7, two periodic gratings were observed. The edge pattern on the film had a maximum depth of 825 nm and a peak-to-peak distance of 63.0 nm (Figure 7D), with an average distance of 2.34 nm (Figure 7B). The center of the pattern had a maximum depth of 343 nm and a peak-to-peak distance of 162 nm (Figure 7E). The pattern spacing was irregularly rippled and became wider at the center and narrower toward the edge. The rippled nanopatterned areas effectively generated iridescence.

**Figure 8:**
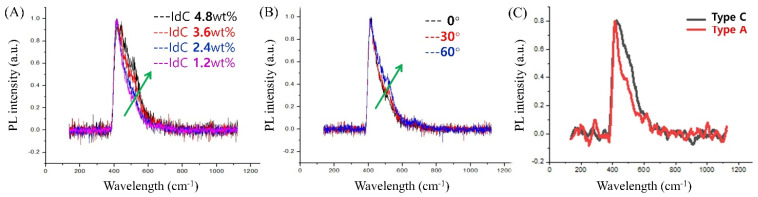
Changes in PL intensity of Alg–IdC films as a function of (**A**) IdC concentration, (**B**) tilt angles in 1.2 wt% IdC and 5 wt% Alg, and (**C**) included ion species.

Photoluminescence (PL) can usually occur after the illumination of a substance with light that has photon energy above the bandgap energy between the valence band (VB) and conduction band (CB). When the unstable electron–hole pair is on the CB, absorbed energy returns to equilibrium in the VB, and the photoluminescence is usually emitted at the corresponding wavelengths. For the Alg–IdC films on quartz, the patterns of their PL intensity peaks in Figure 8A broadened as the IdC concentration increased, while Alg remained constant. Under the condition of the constant concentration of 1.2 wt% IdC based on in 5 wt% Alg hydrogel, the larger the tilt angle of the film, the more the PL intensity pattern increased, as shown in Figure 8B. Regarding the ionic effect, the steric orientation of Alg–IdC containing Ag^+^ ion coordination complexes (Type A) electrons filled in the *4d*-orbital tended to hold more electrons than those containing Ca^2+^ ions as ionic cross-linkers (Type C). Regarding the ionic effect, the conformational orientation of Type A, a coordination complex of Alg–IdC containing Ag^+^ ions filled with electrons in the 4d-orbitals, tended to have more held electrons than Type C containing Ca^2+^ ions as ionic cross-linkers. Therefore, Type A with microspheres began to self-assemble by complexing Ag^+^ ions and was also considered to constitute a narrower PL pattern due to its light-scattering potential than Type C, as shown in Figure 8C. The overall PL intensity increased gradually with increases in the IdC concentration and tilt angle of film. The intensity also increased when silver, rather than calcium, ions were added. 

**Figure 9:**
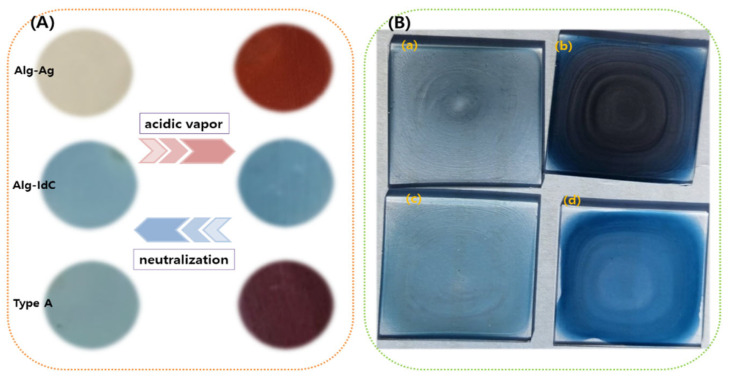
(**A**) Color shift by acidification and neutralization of Alg–Ag complex, Alg–IdC, and Type A based on Alg aqueous solution in desiccator under drying agent. For film containing Ag^+^, the peak red-shifted as it was exposed to acidic vapor. (**B**) Surfaces of (**a**) 2.4 wt% Alg–IdC film Type A containing 0.01 M Ag^+^, (**b**) 4.8 wt% Alg–IdC film containing Type A, (**c**) 2.4 wt% Alg–IdC film Type C containing 0.01 M Ca^2+^, and (**d**) 4.8 wt% Alg–IdC film transferred to a quartz substrate and prepared 1 week before imaging and shown under the sunlight.

Because the blue IdC tends to color-shift in humid air, the blue retention time of Alg–IdC (aq.) at various concentrations was difficult to sustain for more than one month. However, the color of the dry film was preserved for more than one year in a desiccator or refrigerator, as shown in Figure 9A. In contrast, the hue of the sample containing the Ag^+^ composite shifted to wine or red-brown upon exposure to acidic sulfur vapors as a consequence of Ag^+^ salts such as Ag_2_S (black) or AgSO_4_ (black solid) residues, which can originate from pristine materials in the sealing system. The color of the Type A film was darkened by oxidation of complexes upon further exposure to sulfur vapor. At this point, Ag^+^ and even Ag^2+^ as a Lewis acid played a role in the adsorption of Lewis base, sulfuric, or carboxylic groups. The Type A moiety can be used as an indicator or sensor for desulfurization. In Figure 9B, photographs of the distinct rippled patterns on the film after one week of preparation taken under daylight are shown. For surface Type A in Figure 9B(b), the blue color shifted to brown by sulfurization, and the conductivity decreased as the resulting salt was exposed to more air. Because this study only focused on the solubility and coloration based on the type of cations used for film casting, the additional effect of the counter anions corresponding to the cations was not considered. For Type C in Figure 9B(c), the sparse white lines, assumed to be insoluble calcium sulfate salts from IdC, are displayed randomly.

### 3.4. Conductivity of Alg–IdC Materials

The conductivities of the neat Alg–IdC and cross-linked Type C films were similar to those of the natural polymers. For Type A with microspheres, the conductivity was detected to be ca. 7.5–10 kΩ and ca. 100–110 mV at the center on the film versus 2.7 kΩ in 0.01 M AgNO_3_ (*aq.*) at 2 W. Considering a human biocurrent value of 40–60 μA, Type A shows the possibility of being used as a biocurrent smart sensor in a bioelectrolyte medium.

We suggest further investigation into prolonged times for varying coloration, regulation of nano-surface, binding with ions, possibility of optical applications, magnetism, conductivity, metal ion coordination by ligands, complexation with ceramics, and enhancement of physical properties through the chemical structure modification of Alg. We believe that the results of this study will have implications for various fields such as light regulation, eco-friendly camouflage, bird-savers, disposable secured communication, smart coating, color-coded signals, harvesting precious metals, alleviating heavy metal ions in water, and imaging/sensing materials.

## 4. Conclusions

An inspired source of structural coloration was found using innocuous ingredients similar to the ecosystem. The characteristics of the Alg hydrogel were confirmed from the rippled surface with micro-spacing by varying the saturated IdC concentration. Coloration with the bluish Alg–IdC at a concentration gradient was confirmed to have various hues. The UV–vis spectra were obtained at various concentrations and tilt angles. After absorbing Alg–IdC at 280 nm through UV–vis spectroscopy, the optical activity of Alg was expressed as iridescence at a short wavelength in visible light. Alg aqueous solutions containing IdC at various concentrations of 5 wt% or less and a ripped surface pattern on the bluish Alg–IdC film were characterized by intriguing iridescence. The optical properties and effects of structural coloration and scattering properties were also investigated using FT-IR, UV–vis, FE-SEM, AFM, electron microscopy, DSC, TGA, XRD, and spectral characteristics of PL. The surface array of the flexible film had a rippled structure with microspacing. The edge pattern on the film had a maximum depth of 825 nm, a peak-to-peak of 63.0 nm, and an average distance of 2.34 nm. The center of the pattern had a maximum depth of 343 nm and a peak-to-peak of 162 nm. The absorption of UV in the 400–600 nm and 500–700 nm ranges was the same for both the iridescent and non-iridescent surfaces. By adding Ag+ ions to Alg–IdC, self-assembled Type A microspheres were formed, and the conductivity was detected to be ca. 10 μA. Cross-linked bluish Type C was immediately formed upon the addition of Ca2+ ions to the Alg–IdC solution. Type A tends to show red-shift in acidic vapors. It can have applications in fields such as eco-friendly camouflage, disposable anti-counterfeiting, smart displays, and QR code materials for imaging/sensing.

## Figures and Tables

**Table 1:** Thermal characterization of Alg, Alg–IdC, Type A, and Type C using DSC analysis.

Composites	Enthalpy (ΔH) (J/g)	Temperature (°C)	Enthalpy (ΔH) (J/g)	Temperature (°C)
Alg	90.82	120.9	−81.8	161.1
Alg–IdC	71.71	121.5	−79.1	163.9
Type A	−175.1	96.0	−238.6	181.8
Type C	−723.4	106.8	−49.97	174.9

## Data Availability

The data are available upon request from the corresponding authors.

## Supplementary Materials

The following supporting information is available at: https://www.mdpi.com/article/10.3390/polym15173627/s1, Video S1: 90Q; Video S2: Iridescence under sunlight.
